# Editorial: Nano- and Microparticle-Induced Cell Death, Inflammation and Immune Responses

**DOI:** 10.3389/fimmu.2019.00844

**Published:** 2019-04-17

**Authors:** Shrikant R. Mulay, Martin Herrmann, Rostyslav Bilyy, Alexander Gabibov, Hans-Joachim Anders

**Affiliations:** ^1^Nephrologisches Zentrum, Medizinische Klinik und Poliklinik IV, Klinikum der Universität München, Munich, Germany; ^2^Pharmacology Division, CSIR-Central Drug Research Institute, Lucknow, India; ^3^Department of Internal Medicine 3, Friedrich-Alexander-Universität Erlangen-Nürnberg (FAU) and Universitätsklinikum Erlangen, Erlangen, Germany; ^4^Department of Cytology Histology and Embryology, Danylo Halytsky Lviv National Medical University, Lviv, Ukraine; ^5^M. M. Shemyakin and Yu. A. Ovchinnikov Institute of Bioorganic Chemistry of the Russian Academy of Sciences, Moscow, Russia

**Keywords:** inflammation, necrosis, apoptosis, particles, crystals

## Introduction

Crystals, fibers, and other microparticles form inside the body from nutrients or metabolites but also enter the body from outside during environmental or occupational exposures. Altogether crystal and microparticle-related diseases account for numerous medical disorders (1). In some of these disorders, the crystal masses themselves cause the problem, e.g., when stones obstruct the bile ducts or the urinary draining system. In other crystal-immune responses largely account for clinical symptoms and tissue damage, e.g., in gout, silicosis, or asbestosis. This Research Topic in *Frontiers of Immunology* selected a series of articles focusing on how immune and tissue cells respond to crystals, microparticles, and nanoparticles. Our authors shed light on the recent and ongoing research efforts to unravel the molecular mechanisms underlying particle-related tissue injury, inflammation, and remodeling. We hope the readers of this Research Topic find the content useful to further develop the field, to identify common and particle-specific mechanisms of crystallopathies.

## Nano- and Microparticle-Induced Cell Death

Nano- and microparticles induce a broad range of cellular responses when exposed to immune and non-immune cells. These crystalline particles could be either of environmental origin e.g., silica, asbestos, air pollutants, or biological origin e.g., cholesterol, calcium, and monosodium urate (MSU) crystals.

In the current issue, Shu and Shi gave a systematic overview of the physical and chemical properties of solid particles and their host responses. For example, phagocytosis of solid particles is mainly governed by their size, which regulates the inflammatory and cell death responses. Crystals with sharp and pointy edges induce direct injury as compared to spherical crystals and rod-shaped crystals. Accordingly, Köppert et al. also found that primary calciprotein particles, which are spherical, amorphous and soft, were rapidly cleared by liver sinusoidal endothelial cells, whereas secondary calciprotein particles, which are longer, more crystalline, and less soluble, are cleared by Kupffer cells and macrophages.

Cytotoxicity of crystals mainly involves lysosomal rupture, activation of multiple cathepsins, mitochondrial dysfunction leading to either apoptosis or regulated necrosis—viz. mitochondria permeability transition-related necrosis, necroptosis, and pyroptosis (Shu and Shi). In addition, MSU was reported to activate Syk and Src pathways leading to cell death and inflammation in phagocytosis-independent manner (Shu and Shi). Similarly, Shu et al. demonstrated that microscopic cholesterol crystals extract cholesterol from the cell surface upon physical interactions with the plasma membrane, which results in catastrophic plasma membrane rupture and necrosis independent of any known cell death-inducing pathway. Furthermore, in response to stress, a self-cannibalization process termed autophagy is activated, which might be associated with cell death. Frauscher et al. demonstrated that uremia-induced autophagy in vascular smooth muscle cells protects from vascular calcification. Interestingly, pharmacological activation of autophagy protected mice from vascular calcification during chronic kidney disease (CKD).

Ingestion of nano- and microparticles occurs as environmental or occupational pollutants, as well as when used as adjuvants or drug delivery systems. The first line of immune defense—neutrophils regulate immune response to these ingested foreign particles either by removing them by phagocytosis or immobilizing them by trapping with neutrophils extracellular traps (NETs). Silica nanoparticles when ingested during pregnancy cause pregnancy complications. Higashisaka et al. have highlighted the protective roles of neutrophils during pregnancy complications in a murine model of pregnancy. They observed that neutrophil depletion exacerbated silica nanoparticles-induced impairment of placental vessels and increased apoptotic cell death during pregnancy.

## Nano- and Microparticle-Induced Inflammation

A wide range of solid particles is known to induce the formation of NETs (2), which results in the release of intracellular danger associated molecular patterns to initiate the inflammatory response. Bilyy et al. demonstrated that agglomerations of non-stabilized superparamagnetic iron oxide nanoparticles (SPIONs) induce NET formation both *in vivo* and *in vitro*. Interestingly, they observed that inert coating of SPIONs with biocompatible albumin or dextran reduced agglomeration and NET formation by neutrophils and prevented vascular occlusion *in vivo*. These findings again confirm that the physical and chemical properties of solid particles drive host response (Shu and Shi).

At their discovery NETs were considered effective weapons aimed to capture, immobilize, and kill bacteria. Later the list of targets was extended to fungi, viruses, and further pathogens. Recent discoveries have clearly demonstrated that nano-and microparticulate matter is also prone to stimulate NETs formation. The minireview of Li et al. summarizes findings related to NETs formation upon the contact of neutrophils with crystals from MSU, calcium pyrophosphate dihydrate, cholesterol, as well as calcium carbonate and silica. Hydrophobic nanoparticles cause NET formation by membrane disruption and lysosomal leakage. However, for some micro- and nanoparticles the mechanisms of action for the formation of NETs still remains elusive.

Nanocrystals e.g., those from MSU or cholesterol are prone to cluster cell surface receptors to trigger cellular responses. The inhibitory receptors for MSU-induced inflammation CLEC12A and SIRL-1, are thoroughly discussed in the minireview of Fernandes and Naccache. The data suggest that interaction with receptor not only induces mechanical grouping of these cell surface molecules but initiate more complex regulatory processes with distinct effects on leukocyte activation. Targeting inhibitory receptors is a potential therapeutic option to evaluate existing drugs and future drug candidates. However, the list of nanoparticles triggering NET formation can be quite exotic, and very selective.

Cristobalite, contained in volcanic ashes (but not other ash components), was identified as a trigger in macrophages of NLRP3 inflammasome activation with subsequent release of IL-1β (Damby et al.). Inflammasome activation through inhalation of volcanic ash again links air-born microparticles with neutrophil activation and pulmonary diseases. Interestingly, cellular uptake of ash results in lysosomal destabilization, as was reported earlier size dependently for 10 nm diamonds (3), and is followed by activation of mitochondrial responses provoking an oxidative burst.

Calcium oxalate crystals are the naturally occurring particles that reportedly trigger the formation of NETs. Unfortunately, many natural inhibitors of crystal formation like nephrocalcin, osteopontin, uropontin often fail to stop pathological crystal deposition during CKD. The disease is accompanied by fibrosis largely mediated by Transforming Growth Factor beta (TGFβ), and thus anti-TGFβ antibodies are widely used in treatment. Steiger et al. proposed that antibodies can also bind some calcium compounds and thus slow down the process of crystallization in the affected organ. They demonstrated that anti-TGFβ IgG ameliorates CKD not only directly via its influence on the target but also by the prevention of the formation of calcium oxalate crystals in the affected kidneys.

Calcium oxalate nanoparticles were also found to activate human monocyte and enhance local tissue inflammatory responses governed by the production of TNF-a, IL-1b, IL-8, and IL-10. Contact with calcium oxalate crystals but not with zinc oxide nanoparticles stimulated macrophage differentiation into the inflammatory M1-type (Dominguez-Gutierrez et al.). In addition, the properties of a putative calcium oxalate receptor are discussed in the paper.

Another natural defense mechanism against nanoparticles was reported in the work (Marschner et al.), where long pong pentraxin 3 (PTX3), an opsonin known to interact with dead cells and other extracellular microparticles, was identified as an endogenous factor inhibiting growth of calcium oxalate crystals. By limited CaOx crystal aggregation and adhesion to tubular cell membranes it serves as one of several endogenous inhibitors of stone formation in nephrocalcinosis and potentially other crystallopathies.

## Nano- and Microparticle-Induced Immune Responses

When it comes to immune response the first task of the immune system is to recognize the invader, and micro-and nanoparticles are hard to be recognized, since usually they are the same compounds the body encounters daily in smaller concentrations or in a different soluble form (e.g., MSU). The review of Nakayama summarizes recent advances in our understanding how macrophages recognize crystals particles: some crystal particles are negatively charged and are recognized by scavenger receptor family members in a charge-dependent manner. Alternatively, a model for receptor-independent phagocytosis of crystals has also been proposed to explain their clearance by macrophages (Mahon and Dunnet). Effect of the nanoparticle engulfment can be quite different. Silver and gold nanoparticles when covered with tannic were able to induce maturation of dendritic cells and stimulate the uptake of viral particles (HCV-2) (Orlowski et al.).

Unfortunately, there are much more of them causing harmful effects. The minireview by Mahon and Dunne summarize the role of particles associated with gout, calcium pyrophosphate deposition (CPPD) disease, and osteoarthritis (OA). As well as wear-debris particles generated from prosthetic implants and contributing to joint destruction through the production of cartilage-degrading enzymes and pro-inflammatory cytokines, driving periprosthetic osteolysis which impacts on the longevity of total joint replacements. Understanding the danger of implanted material will allow us to better define molecular targets to inhibiting side effects of implantation-associated dangerous micro and nanoparticles (Chulpanova et al.; Orlowski et al.).

Some nanoparticles like silica are known for decades to cause disease and autoimmune disorder, but it is not that easy to study the conditions in experimental setup due to the fact that most established animal (mice) cell line are prone to develop only a part of the whole repertoire of interactions attributable to autoimmune conditions in human. In the work of Mayeux et al. this limitation was overcome by in-depth analysis of diversity outbred mice, revealing not only association of silicosis with lung autoimmune markers, bronchoalveolar lavage fluid cells, IL-6 and anti-ENA5, but also stronger silica-induced inflammation between mal mice. We should remember that cellular remnants and “apoptotic blebs” are also of nano- and microscale, and usually engulfment is silent and anti-inflammatory process (Tucher et al.). Apoptotic cells were known to produce different type of micro and nano extracellular vesicles (EV) they were known to be differently glycosylated and distinctly processed by macrophages (4). However, the formation of nanoparticles, like exosomes and others extracellular vesicles not related to apoptosis make their discrimination complicated. Group of Schiller have demonstrated that protein composition of apoptotic EV is quite unique and thus can serve as reliable discrimination factor; some danger signals like HMGB1 protein could be found only in apoptotic-related microvesicles (Tucher et al.). Another important finding suggests that release of large extracellular vesicles, 200 to 1,000 nm in diameter, involve proteasome action (Tucher et al.).

But when it comes to cancer, it looks that EV are becoming powerful communicating and instructing tools. In minireview “Role of macrovesicles produced by cancer cells,” immune cells and mesenchymal stem cells are discussed in details, demonstrating how modification of the extracellular vesicle cargo can target specific tumor mechanisms responsible for tumor formation and progression to develop new therapeutic strategies and to increase the efficacy of antitumor therapies (Chulpanova et al.).

Immunosuppression is a key factor in silent clearance of dying cell microdebris, thus it's role in dealing with disease associated nano- and micro particles is crucial. The review by Huaux details the contribution of immunosuppressive cells and their derived immunoregulatory mediators and discusses the role of inflammatory vs. immunosuppressive mechanisms connecting micro- and nano-particles with pathogenesis of chronic diseases. The review summarizes role of immunosuppressive cytokines, involvement of regulatory T- and B lymphocytes, immunosuppressive myeloid cells and discusses how particle-related diseases can develop independently of chronic inflammation, enriches current bioassays predicting particle toxicity and suggests new clinical strategies for treating patients affected by particle-associated diseases (Marschner et al.).

## Perspectives

The studies of this issue illustrate that crystals and microparticles induce diverse biological responses beyond triggering the NLRP3 inflammasome and the secretion of IL-1β from myeloid cells (5). Crystal biology just started as an interdisciplinary field and certainly holds bright promises for major discoveries also in the future. We still do not understand the full spectrum of molecular mechanisms shared across particles of different natures as well as different sizes and shapes. Often molecular machinery prone to recognize cellular debris can also react with inorganic crystals and vice versa. We lack an understanding of the particle-specific immune responses. Another unsolved issue is the functional differences between microparticle-induced neutrophil necroptosis and NET formation, a regulated form of chromatin release different from regulated neutrophil necrosis (6, 7). Only an interdisciplinary approach can address these important questions and eventually lead to new treatment modalities for patients with crystal- and microparticle-related disorders.

## Author Contributions

All authors listed have made a substantial, direct and intellectual contribution to the work, and approved it for publication.

### Conflict of Interest Statement

The authors declare that the research was conducted in the absence of any commercial or financial relationships that could be construed as a potential conflict of interest.
